# Time utilization and perceived psychosocial work environment among staff in Swedish primary care settings

**DOI:** 10.1186/s12913-018-2948-6

**Published:** 2018-03-07

**Authors:** Eva Anskär, Malou Lindberg, Magnus Falk, Agneta Andersson

**Affiliations:** 1 Department of Medical and Health Sciences, Linköping University, Linköping, Sweden; 2 Primary Health Care Centre in Mantorp, and Department of Medical and Health Sciences, Linköping University, Mantorp, Sweden; 3 1177 Medical Advisory Service, Linköping, Sweden; 4 Research and Development Unit, and Department of Medical and Health Sciences, Linköping University, Linköping, Sweden

**Keywords:** Work-time allocation, Primary care, Occupational health, Organization and administration, Stress

## Abstract

**Background:**

Over the past decades, reorganizations and structural changes in Swedish primary care have affected time utilization among health care professionals. Consequently, increases in administrative tasks have substantially reduced the time available for face-to-face consultations. This study examined how work-time was utilized and the association between work time utilization and the perceived psychosocial work environment in Swedish primary care settings.

**Methods:**

This descriptive, multicentre, cross-sectional study was performed in 2014–2015. Data collection began with questionnaire. In the first section, respondents were asked to estimate how their workload was distributed between patients (direct and indirect patient work) and other work tasks. The questionnaire also comprised the Copenhagen Psychosocial Questionnaire, which assessed the psychosocial work environment. Next a time study was conducted where the participants reported their work-time based on three main categories: direct patient-related work, indirect patient-related work, and other work tasks. Each main category had a number of subcategories. The participants recorded the time spent (minutes) on each work task per hour, every day, for two separate weeks. Eleven primary care centres located in southeast Sweden participated. All professionals were asked to participate (*n* = 441), including registered nurses, primary care physicians, care administrators, nurse assistants, and allied professionals. Response rates were 75% and 79% for the questionnaires and the time study, respectively.

**Results:**

All health professionals allocated between 30.9% - 37.2% of their work-time to each main category: direct patient work, indirect patient work, and other work. All professionals estimated a higher proportion of time spent in direct patient work than they reported in the time study. Physicians scored highest on the psychosocial scales of quantitative demands, stress, and role conflicts. Among allied professionals, the proportion of work-time spent on administrative tasks was associated with more role conflicts. Younger staff perceived more adverse working conditions than older staff.

**Conclusions:**

This study indicated that Swedish primary care staff spent a limited proportion of their work time directly with patients. PCPs seemed to perceive their work environment in negative terms to a greater extent than other staff members. This study showed that work task allocations influenced the perceived psychosocial work environment.

**Electronic supplementary material:**

The online version of this article (10.1186/s12913-018-2948-6) contains supplementary material, which is available to authorized users.

## Background

Over the past few decades, reorganizations and structural changes in the Swedish health care system have affected how health care professionals utilize their time. One example is the implementation of electronic information technology, such as electronic patient records. When combined with a reduced number of health care administrators, this change has led to more administrative work tasks for health care professionals [1]. Consequently, the time available for face-to-face consultations has decreased. An international comparison showed that, compared to primary care physicians (PCPs) of other nations, Swedish PCPs devoted fewer working hours face-to-face with patients [2].

In a Swedish Health Policy survey, most PCPs were dissatisfied with the amount of time they could devote to patients, and they rated their job as very or extremely stressful [2]. Complex, stressful working conditions have also been reported among nurses in primary care [3, 4]. A recent systematic review concluded that improving the psychosocial work environment might prevent stress-related disorders from occurring among workers in several workplaces, including the health care sector [5]. A Swedish study stated that role conflicts are important predictors of job dissatisfaction in the health care sector, and consequently, in the psychosocial work environment [6].

Several studies have shown that the skills and competences of health care professionals in primary care have been underutilized [7, 8]. For example, registered nurses (RNs) and PCPs perform work tasks that can be performed by care administrators or health technicians [8]. Dissatisfaction with the work situation increases the risk of staff leaving their jobs and seeking new positions. Indeed, retaining staff is an ongoing challenge for Swedish health care.

To our knowledge, there is a shortage of studies on time utilization and on the psychosocial work environment in primary care, particularly studies that include all staff categories. From a managerial perspective, it is important to know how much time staff members spend on different work tasks; this information can be used to optimize clinical efficiency, ensure work satisfaction, and facilitate staff retention.

The aims of this study were to investigate work-time utilization among different professionals in Swedish primary care and to explore associations between work-time utilization and the psychosocial work environment.

## Methods

A descriptive, multicentre, cross-sectional study was performed in primary care institutions in southeast Sweden.

### Setting

Sweden has nearly 10 million inhabitants. The increasing proportion of older individuals in the population presents a major challenge for the health care system, as in other northern European countries [9]. In Sweden, health care is publicly funded. The health care organization is managed by 21 county councils/regions. Out of the county councils 13 have an extended responsibility for regional development and are therefore named regions, all will subsequently be called county councils. The county councils are responsible for delivering both hospital care and primary care. Sweden has a total of seven university hospitals, 70 county council-driven hospitals (six are private), and approximately 1200 primary care centres [10] including private primary care centres contracted by the county councils. Most primary care centres are open during office hours.

### Participants

This multicentre study included four county councils (Region Östergötland, Region Jönköping, Kalmar county council, and Södermanland county council), which served approximately 1.3 million inhabitants in southeast Sweden. Among the 151 primary care centres in this geographic area, this study selected 23 primary care centres, based on purposive sampling [11]. The goal was to capture a wide range of perspectives, including for example, the centre size, geographical location, and urban or rural setting. The managers of these 23 primary care centres were contacted and informed about the study. The study was approved by the managers of 11 primary care centres (ten public and one private). These primary care centres were located in both rural (*n* = 5) and urban (*n* = 6) areas and varied in size; the smallest had 20 employees and the largest had 81 employees. All professionals (*n* = 441 individuals) were invited to participate, including RNs, PCPs, care administrators, nurse assistants (NAs), and allied professionals (physiotherapists, occupational therapists, psychologists, counsellors, dieticians, chiropodists). The PCP group consisted of general practitioners and physicians in training. The employees at each primary care centre were informed about the study at a staff meeting. They also received written information.

### Data collection

A questionnaire was distributed to all staff members at each primary care centre by e-mail with the web-based tool, Publech Survey 5.7. One reminder was sent after 2 weeks. In the first section of the questionnaire, participants were asked to estimate the proportions of time spent on work tasks that involved patients (directly and indirectly) and time spent on other work. Examples of work tasks were given for each of these categories (i.e., direct, indirect, and other). Also, participants assessed the psychosocial work environment with the validated instrument, the Copenhagen Psychosocial Questionnaire (COPSOQ) [12–16]. The COPSOQ is constructed to allow researchers to select the scales appropriate for the aim of the survey. For this study, the selected scales covered six areas of interest: quantitative demands (4 items), stress (4 items), role conflicts (4 items), quality in work (3 items), conflicts between work and personal life (4 items), and positive impact of work on personal life (2 items). The two scales called ‘quality in work’ and ‘positive impact of work on personal life’ were not part of the original COPSOQ, but they were added by the creators of the COPSOQ for inclusion in studies conducted in the health care sector. The questionnaire also included a section with questions regarding illegitimate tasks [17]; that analysis will be reported elsewhere.

Next, a time study was conducted with a form (delivered on paper or digitally) that was developed specifically for this study (Additional file 1). Participants used the form to record the time (min) they spent on each work task, every hour, every day, over two separate weeks, Monday to Friday, during office hours. The form contained three main categories (called work tasks) and a number of subcategories for each main category. For example, the first main category was *direct patient-related work tasks*, and it included face-to-face contacts with patients and telephone contacts with patients or their next of kin. The second main category was *indirect patient-related* work tasks, and it included documentation of patient data, signing journal entries, prescribing medical drugs, and entering data into different health care records databases and quality registries. The third main category was *other work tasks*, and it included meetings with colleagues, continuing education, e-mail management, managing equipment and facilities, dealing with computer problems, waiting time, other writing/administrative tasks, and pauses (short breaks between tasks, such as a brief coffee break). Prior to the main study, the form was validated by two experts (a PCP and a RN), and minor adjustments were made. The participants were given a pamphlet with instructions on how to complete the form. A total of 202 office hours were excluded from the analyses, due to incorrect reporting or illegibility; these were classified as internal drop-outs.

Due to the large amount of administrative work tasks in Swedish primary care [2], administrative work tasks were divided into patient-related tasks and organization and service-related tasks. Patient-related administration included tasks like documentation, dictation, scheduling appointments, signing journal entries, referral management, handling mail, prescribing medical drugs, entering data into health care records and quality registries, and prescribing medical aids. Organization-related administration and services included tasks like meetings at the work place, other writing tasks/administration, managing equipment and facilities, e-mail management, meetings outside the work place, scheduling, managing computer problems, ordering medical supplies, including laundry, and non-patient-related telephone contacts.

### Statistical analysis

#### COPSOQ

Descriptive statistics were performed to calculate mean scores and standard deviations (SD). An item with five response alternatives was scored from 0 to 100, i.e. 0, 25, 50, 75, and 100 and a four-response item was scored 0, 33.3, 66.7, and 100. The standardized scores facilitated comparisons between different scales. The total score for a scale was calculated as the mean of the scores for the individual items in that scale. A difference of 5 in the mean value was defined as a clinically significant change for each scale [16]. A high score on the scales ‘quantitative demands’, ‘stress’, ‘role conflicts’, and ‘conflicts between work and personal life’ indicated a negative psychosocial work environment. A high score on the scales ‘quality in work’ and ‘positive impact of work on personal life’ indicated a positive psychosocial work environment.

#### Time-study

Response rates for study participation are expressed as the proportion of responses for each section of data collection. The responses were also subcategorized by profession and age. Age is expressed as the mean, range (min-max), and standard deviation (SD), for each profession and for the entire study sample. The mean estimated proportions of time spent on work tasks were compared to self-reported time use (based on the time study) with the paired t-test. Descriptive statistics of work tasks are reported as the mean percentage and the min-max. The means and SD of COPSOQ scales were compared between professions with the analysis of variance (ANOVA) and post-hoc Tukey test. Pearson’s *r* correlation was used to analyse associations between COPSOQ scales and the proportions of time spent on different work tasks and associations between COPSOQ scales and age. A two tailed *p*-value ≤0.05 was considered statistically significant. Statistical analyses were performed with the Statistics Package for Social Sciences (SPSS) version 22.

Data collection was carried out from March 2014 to February 2015.

#### Ethics

This study was approved by the Ethics Review Board in Linköping, Sweden (D.nr. 2014/81–31). All data material was stored in a database with a high level of security that could only be accessed by the authors. Participants received information about the study verbally at a staff meeting and also by written information at the start of the data collection. Participants were informed that the study was voluntary, that they could drop out of the study without explanation at any time, and that confidentiality was guaranteed. Participants agreed to participate by responding to the questionnaire and time study.

## Results

Overall, of 441 individuals invited to participate, 391 took part in the study; thus, the response rate was 89%. However, the response rates were different for the different types of data collection instruments (Table 1). The majority of participants were women. Between 88 to 100% of participants were women in all professional categories, except in the group of PCPs, which included 55% women.

**Table 1:** The professions and mean ages of participants in the entire study (study sample), and the numbers of individuals in each profession that completed each study section

	Study sample				Self-estimation of work time	Questionnaire PWE^a^	Time study	Questionnaire PWE and time study
Professions	n (%)	Mean age, years	(min-max)	(SD)	n	n	n	n
Registered nurse	148 (38)	52	(22–67)	(9.6)	129	127	139	118
Physician	86 (22)	46	(28–70)	(11.7)	63	63	75	52
Care administrator	70 (18)	49	(26–66)	(11.2)	66	65	61	56
Nurse assistant	44 (11)	54	(33–67)	(8.7)	35	35	42	33
Allied professions	43 (11)	47	(34–65)	(12.4)	40	39	33	29
Total sample (All professions)	391 (100)	50	(22–70)	(10.9)	333	329	350	288

^a^Psychosocial Work Environment

The estimated proportions of time spent on work tasks differed from the self-reported time use recorded in the time study. All professionals estimated that they spent a greater proportion of time on direct patient work tasks (Table 2) than the proportion recorded in the time study. Conversely, the estimated proportion of time spent on other work tasks was lower than the proportion recorded in the time study (Table 3).

**Table 2:** Comparisons between self-estimated and self-reported proportions of time spent on work tasks

		Direct patient-related work tasks					Indirect patient-related work tasks					Other work tasks			
		Self-assessed	Self-reported				Self-assessed	Self-reported				Self-assessed	Self-reported		
Professions	*n*	%	%	CI for difference in mean	*p*	*n*	%	%	CI for difference in mean	*p*	*n*	%	%	CI for difference in mean	*p*
Registered nurse	120	54.5	42.2	9.5–15.2	< 0.001	119	27.6	27.2	−2.1-2.8	0.750	120	18.7	30.9	14.4–9.9	< 0.001
Physician	52	42.9	34.4	4.7–12.3	< 0.001	52	35.7	32.3	0.3–6.4	0.031	52	21.3	33.3	17.1–6.9	< 0.001
Care administrator	49	22.8	20.3	−1.7–6.6	0.238	56	57.9	44.8	7.3–18.8	< 0.001	57	21.3	38.5	22.6–11.8	< 0.001
Nurse assistant	33	53.5	40.2	7.3–19.3	< 0.001	33	22.5	18.5	−0.7-8.7	0.092	33	22.6	41.4	22.8–14.8	< 0.001
Allied professionals	31	58.0	40.5	11.0–24.1	< 0.001	31	22.4	27.0	−8.6-0.6	0.025	31	16.8	32.5	21.1–10.3	< 0.001
Overall	285	47.2	36.6	8.7–12.5	< 0.001	291	33.7	30.5	1.4–5.0	< 0.001	293	19.9	34.2	16.0–12.4	< 0.001

Paired t-test

**Table 3:** Proportions of time spent on main categories and subcategories of work tasks, for each profession based on self-reported data from the time study

Work tasks	Overall			Registered nurse			Primary care physician			Care administrator			Nurse assistant			Allied professions		
	*n*	%	(min–max)	*n*	%	(min–max)	*n*	%	(min–max)	*n*	%	(min–max)	*n*	%	(min–max)	*n*	%	(min–max)
*Direct patient-related work tasks*	342	37.2	(0.1–84.0)	139	42.6	(2.0–84.0)	75	35.9	(3.8–61.1)	53	19.9	(0.1–66.4)	42	40.4	(15.7–83.0)	33	40.8	(21.0–67.5)
Face-to-face contact with patients		73.1	(0–100)		55.2	(0–98.3)		81.8	(63.7–100)		84.6	(0–100)		90.0	(24.4–100)		88.8	(71.9–100)
Telephone contact with patients		22.4	(0–100)		39.8	(0–100)		15.9	(0–31.4)		8.5	(0–100)		4.6	(0–19.9)		9.2	(0–22.8)
Telephone contact with the patient’s next of kin		2.0	(0–100)		2.8	(0–19.1)		1.1	(0–9.2)		3.1	(0–100)		0.5	(0–3,2)		0.8	(0–4.6)
Remaining tasks		2.5	(0–66.5)		2.2	(0–45.4)		1.2	(0–18.6)		3.8	(0–55.6)		5.0	(0–66.5)		1.3	(0–14.2)
*Indirect patient-related work tasks*	348	30.9	(0.1–88.7)	138	27.6	(1.4–56.4)	75	34.1	(6.5–62.6)	60	45.3	(0.1–88.7)	42	18.2	(0.8–46.1)	33	27.8	(14.8–51.9)
Documentation in health care records, ordering tests		45.9	(0–100)		51.6	(12.5–100)		11.9	(0–59.6)		76.4	(0–100)		41.0	(0–94.0)		49.9	(11.4–83.2)
Reading health care records		11.8	(0–58.8)		13.3	(0–58.8)		16.8	(0.5–46.6)		0.9	(0–26.8)		8.6	(0–39.8)		17.8	(0–53.2)
Contact with other caregivers about patient cases		8.2	(0–100)		9.6	(0–37.8)		9.8	(0–31.5)		4.3	(0–100)		8.1	(0–60.7)		5.7	(0–26.7)
Dictation		6.1	(0–75.1)		1.0	(0–19.6)		24.0	(4.1–75.1)		0.6	(0–21.9)		0.7	(0–11.4)		3.7	(0–35.8)
Scheduling appointments		5.6	(0–100)		6.8	(0–50.0)		0.9	(0–4.7)		5.5	(0–100)		9.8	(0–100)		6.3	(0–46.9)
Signing journal entries		4.3	(0–55.7)		3.2	(0–19.6)		13.0	(0–55.7)		0.03	(0–1.1)		0.4	(0–4.9)		1.7	(0–15.3)
Referral management		2.6	(0–21.9)		1.5	(0–14.4)		5.5	(0–15.3)		1.7	(0–16.2)		0.9	(0–8.1)		4.0	(0–21.9)
Handling mail		2.2	(0–100)		1.3	(0–41.7)		2.8	(0–13.8)		3.8	(0–100)		2.6	(0–32.6)		0.9	(0–10.6)
Prescribing medical drugs		2.2	(0–29.2)		0.4	(0–6.5)		9.2	(0–29.2)		0.3	(0–5.7)		0.04	(0–1.6)		0.4	(0–6.3)
Entering data into health care records and quality registries		1.2	(0–35.3)		2.1	(0–35.3)		0.2	(0–2.7)		0.6	(0–8.5)		1.3	(0–24.2)		1.0	(0–18.3)
Drug management		1.1	(0–35.8)		2.0	(0–35.8)		0.8	(0–10.5)		0.4	(0–17.1)		0.4	(0–5.1)		0.03	(0–1.0)
Patient-related transport		1.1	(0–26.7)		1.7	(0–26.7)		0.8	(0–8.4)		0.0	(0–0)		1.2	(0–23.6)		1.0	(0–16.5)
Prescription of medical aids		0.9	(0–25.4)		1.9	(0–25.4)		0.3	(0–13.1)		0.0	(0–0)		0.4	(0–10.7)		0.2	(0–2.3)
Contact with authorities		0.6	(0–18.3)		0.5	(0–7.9)		0.7	(0–8.3)		0.3	(0–8.8)		0.2	(0–3.2)		1.7	(0–18.3)
Remaining tasks		6.4	(0–100)		3.2	(0–34.4)		3.2	(0–40.8)		5.2	(0–73.5)		24.5	(0–100)		5.7	(0–54.9)
*Other work tasks*	350	32.9	(3.5–99.3)	139	30.0	(3.5–98.0)	75	30.0	(11.1–89.6)	61	38.2	(9.0–99.3)	42	41.4	(14.1–77.0)	33	31.4	(6.1–52.4)
Meetings at the work place		21.0	(0–77.1)		23.4	(0–67.9)		24.6	(0–64.3)		18.7	(0–77.1)		14.2	(0–35.7)		16.1	(0–59.4)
Pauses		19.7	(0–100)		23.4	(0–78.5)		13.7	(0–42.4)		21.6	(0–65.7)		16.0	(4.3–35.8)		18.7	(1.2–100)
Other writing tasks/administration		15.9	(0–84.2)		14.4	(0–51.8)		6.3	(0–84.2)		32.4	(0–79.8)		16.8	(0–40.0)		11.7	(0–51.0)
Continuing education		10.2	(0–100)		8.3	(0–62.8)		21.6	(0–90.9)		4.8	(0–100)		2.4	(0–55.2)		12.9	(0–61.9)
Managing equipment and facilities, non-computer related		6.5	(0–55.6)		5.6	(0–41.4)		0.4	(0–10.1)		1.6	(0–17.9)		27.8	(0–55.6)		6.1	(0–38.8)
Managing e-mails		5.5	(0–47.0)		6.1	(0–26.9)		4.6	(0–15.9)		5.1	(0–26.5)		4.3	(0–16.2)		7.7	(0–47.0)
Receiving and performing mentoring		3.5	(0–63.7)		2.1	(0–36.2)		10.0	(0–63.7)		0.5	(0–7.5)		0.8	(0–8.4)		3.6	(0–41.1)
Meetings outside the work place		3.4	(0–61.3)		3.3	(0–33.1)		4.6	(0–61.3)		2.4	(0–21.3)		1.6	(0–19.2)		5.9	(0–37.4)
Waiting, non-computer related		2.0	(0–37.3)		2.4	(0–23.6)		1.6	(0–21.5)		0.4	(0–9.3)		3.6	(0–37.3)		1.9	(0–20.3)
Scheduling		1.6	(0–57.1)		1.4	(0–18.2)		2.2	(0–57.1)		2.0	(0–40.5)		1.1	(0–11.2)		0.9	(0–7.7)
Managing computer problems		1.3	(0–28.3)		1.0	(0–22.2)		1.5	(0–28.3)		2.2	(0–20.8)		1.1	(0–6.7)		0.9	(0–8.8)
Non-patient-related transport		0.9	(0–18.3)		0.8	(0–14.3)		1.0	(0–15.3)		0.2	(0–6.4)		0.5	(0–12.1)		2.5	(0–18.3)
Ordering medical supplies, including laundry		0.7	(0–23.0)		0.3	(0–6.5)		0.01	(0–0.5)		0.6	(0–23.0)		3.9	(0–12.1)		0.4	(0–5.6)
Non-patient-related telephone contacts		0.6	(0–20.5)		0.6	(0–9.0)		0.5	(0–20.5)		0.6	(0–6.0)		0.5	(0–3.0)		0.9	(0–13.6)
Remaining tasks		7.3	(0–40.0)		7.1	(0–40.0)		7.5	(0–37.8)		7.1	(0–27.5)		5.6	(0–26.0)		10.0	(0–38.9)

The main categories have been marked with italics

The time study was completed by 350 of the 441 invited individuals. Thus, the response rate among primary care centres was 79% (range, 59–94%; Table 1). Over one million minutes were reported (1,113,879 min, lunch breaks excluded) over the 2 weeks included in the time study. Direct patient work tasks required 37.2%; indirect patient work tasks required 30.9%; and other work tasks required 32.9% of the total work-time. The dominant indirect patient work task was documentation (45.9% of the time). RNs had the largest share of direct patient work tasks (42.6%), followed by allied professionals (40.8%), NAs (40.4%), and PCPs (35.9%). However, PCPs spent 81.8% of their direct patient work time on working face-to-face with patients. In contrast, RNs spent 42.6% of their direct patient work time on the telephone with patients or the patient’s next of kin. Care administrators had the largest share of indirect patient work tasks (45.3%), followed by PCPs (34.1%). NAs had the largest share of the other work tasks (41.4%), compared to PCPs, RNs, and allied professionals. Overall, pauses constituted about one fifth of the other work tasks for all groups, except PCPs (13.7%). Thus, pauses constituted 6.5% of the total work-time, but the percentage varied among different professions, as follows: PCPs (4.1%), RNs (7.0%), allied professionals (5.8%), NAs (6.6%), and care administrators (8.3%; Table 3).

Over 41% of the total work-time was spent on administrative and service work tasks. This percentage included 22.9% for patient-related administration and 19.4% for organization-related administration and service (Table 4).

**Table 4:** Proportions of time spent on administrative and service work tasks, by profession

Profession	Patient-related administration^a^		Organization-related administration and service^b^		Total administration and service^a,b^	
	*n*	%	*n*	%	*n*	%
Registered nurse	138	18.9	139	17.3	138	35.7
Primary care physician	75	22.9	75	12.5	75	35.4
Care administrator	58	43.5	60	27.5	57	68.1
Nurse assistant	41	10.3	42	29.8	41	40.3
Allied professionals	33	18.9	32	16.0	32	34.4
Overall	345	22.9	348	19.4	343	41.5

^a^Patient-related administration tasks included: documentation, dictation, administering appointments, signing journal entries, referral management, handling mail, prescribing medical drugs, entering data into health care records and quality registries, and prescribing medical aids

^b^Organization-related administration tasks and service included: meetings at the work place, other writing tasks/administration, managing equipment and facilities, e-mail management, meetings outside the work place, scheduling, managing computer problems, ordering medical supplies, including laundry, and non-patient-related telephone contacts

### Psychosocial work environment

The mean COPSOQ scores, according to profession, are shown in Table 5. Compared to reference values (available for four of the six scales), for all professionals, the mean scores for quantitative demands and stress were five scale-steps above the threshold. For role conflicts, the score was under the threshold (low values indicated a positive psychosocial work environment). PCPs reported higher scores for quantitative demands, stress, role conflicts, and conflicts between work and personal life, compared to other professionals (Table 5). The mean scores for role conflicts and conflicts between work and personal life were significantly different between PCPs and all other professionals (Additional file 2).

**Table 5:** Scores for psychosocial factors measured with the COPSOQ questionnaire, according to profession

Professions	*n*	Quantitative demands^b^		Stress^b^		Role conflicts^b^		Quality in work^c^		Conflicts between work and personal life^b^		Positive impact of work on personal life^c^	
		Mean	SD	Mean	SD	Mean	SD	Mean	SD	Mean	SD	Mean	SD
Registered nurse	124	47.7	19.4	31.9	18.0	25.0	17.7	77.2	12.3	28.0	25.7	57.7	23.4
Primary care physician	63	61.1	22.1	41.2	19.1	37.2	18.2	78.2	11.9	49.2	31.4	59.0	26.8
Care administrator	63	44.3	17.9	32.2	19.4	24.8	19.9	78.6	11.7	18.1	19.3	51.6	27.6
Nurse assistant	35	34.8	13.3	27.3	17.0	22.5	18.7	80.0	15.4	14.8	16.4	56.7	25.3
Allied professionals	39	51.3	20.9	32.9	19.6	24.7	18.4	78.4	14.0	30.6	28.9	62.8	23.4
Overall	324^a^	48.7	20.6	33.4	18.9	27.0	19.0	78.1	12.6	29.1	27.6	57.3	25.2
Reference value		40.2		26.7		42.0		^d^		33.5		^d^	

All scores are expressed as the mean and standard deviations (SD); scores were transformed to a scale of 0 to 100

^a^Participants that did not answer all questions were excluded

^b^Low value is a positive rating

^c^High value is a positive rating

^d^Reference value not available

We analysed correlations between scales in the psychosocial work environment and time allocations. For allied professionals, the strongest correlation was between role conflicts and the proportion of time spent on total administration and service tasks. Thus, the more time one spent on administration and service work tasks, the more role conflicts reported. Similarly, among RNs, a correlation was observed between role conflicts and the proportion of time spent on direct patient work tasks. Thus, the less time spent on direct patient work tasks, the more role conflicts reported.

We also analysed whether stress was related to age. The strongest correlation between age and stress was observed among NAs; in that group, the younger the NA, the more stress reported (Table 6).

**Table 6:** Correlations between COPSOQ scores and proportions of time spent on work tasks and age^a^

		Role conflicts and Total administration and service			Role conflicts and Patient-related administration			Quantitative demands and Total administration and service			Role conflicts and Direct patient-related work tasks			Role conflicts and Age			Stress and Age	
Professions	*n*	*r-factor*	*p-value*	*n*	*r-factor*	*p-value*	*n*	*r-factor*	*p-value*	*n*	*r-factor*	*p-value*	*n*	*r-factor*	*p-value*	*n*	*r-factor*	*p-value*
Allied professionals	29	0.566	0.001	29	0.432	0.019	29	0.168	0.385	29	−0.193	0.315	39	0.394^b^	0.013	39	−0.106	0.519
Primary care physician	52	0.299	0.031	52	0.199	0.157	52	0.293	0.035	52	−0.093	0.511	63	0.081	0.526	63	0.254^c^	0.045
Registered nurse	116	0.110	0.242	116	0.020	0.829	117	0.128	0.169	117	−0.184^d^	0.047	125	0.212	0.018	125	0.132	0.143
Care administrator	51	0.144	0.314	52	−0.128	0.365	52	−0.183	0.195	47	0.207	0.163	63	0.192	0.132	63	0.105	0.412
Nurse Assistant	32	0.070	0.702	32	0.084	0.646	32	0.235	0.195	33	0.004	0.984	35	0.357	0.035	35	0.425	0.011
Overall	280	0.050	0.403	281	0.038	0.531	282	−0.027	0.657	278	−0.080	0.183	325	0.245	< 0.001	325	0.175	0.002

^a^Pearson’s correlation; ^b^The younger the staff member, the more role conflicts reported; ^c^The younger the staff member, the more stress reported; ^d^The less time spent on direct patient work tasks, the more role conflicts reported

## Discussion

This study illustrated the fact that primary care staff appeared to spend a considerable proportion of work-time on indirect patient work tasks and other, non-patient related work tasks. Just above one third of the work-time was spent on work tasks associated with direct patient contact. PCPs reported a higher degree of negativity in the psychosocial work environment than other staff groups. Among PCPs and allied professionals, a positive correlation was observed between role conflicts and the proportion of total time spent on administration and service. That is, the more time spent on administrative and service work tasks, the more role conflicts they reported. Similarly, for RNs, we found a negative correlation between the proportion of time spent on direct patient work tasks and the degree of role conflicts. That is, the less time spent on direct patient work tasks, the more role conflicts reported. Younger staff in all professions reported a higher degree of negativity in the work environment compared to older staff. PCPs reported the lowest proportion of time spent in pauses, which might reflect a stressful work situation.

The PCPs, allied professionals, RNs, and care administrators reported high values for quantitative demands and stress, indicating a perception of adverse working conditions. The work situation in primary care is often characterized as demanding and complex [4, 18, 19], and adverse psychosocial work conditions among primary care staff can be associated with a poor quality of life [20]. Our results indicated that a high administrative workload had a negative impact on the reported psychosocial work environment. This finding was consistent with previous research, which showed that predominantly administrative and bureaucratic organizations were associated with heightened levels of job dissatisfaction [21]. One explanation for the finding that the administrative workload had a negative impact on perceived role conflicts could be that the amount of administrative work was unexpected and unwanted, and it did not reflect the university curriculum description for medical staff. The results of our study confirmed that Swedish PCPs, in general, spent a considerable amount of time on administration, and this factor could be hindering efficient patient care [22]. RNs and allied professionals also spent a considerable amount of time on administrative work tasks, which may have a negative effect on patient care, due to the lower proportion of time spent face-to-face. Nevertheless, documentation has several positive aspects; it is an important tool for achieving high quality care. Previous research has shown that successful care delivery depended on the fact that nurses valued face-to-face interactions with patients [23]. That finding was consistent with our results; we found that the less time spent on direct patient work tasks, the more reports of role conflicts. In conclusion, competence in primary care could be improved by transferring some administrative and service tasks, mainly organizational, to other staff categories [8, 22, 24]; e.g., to professional administrators or a service staff. However, some administrative work tasks can only be performed by medical staff; e.g., signing journal entries, dictation, referral management, and prescribing medical drugs and medical aids.

All health care professional in this study overestimated the proportion of time spent in direct patient work tasks, compared to the results from the time study. This might reflect the high value that medical staff placed on direct patient contact, as shown by Bringsén et al. [23]*.* Our results also showed that RNs spent a substantial amount of time on telephone consultations, as part of direct patient work. This was not surprising, considering that, in Swedish primary care, telephone accessability is a prioritized work task for RNs, as a result of political decisions.

In contrast to other professionals in Swedish primary care, allied professionals rarely have colleagues in the same profession at primary care centres. Therefore, they lack an interactive work environment, where they can spontaneously discuss issues with peers, and they must solve most administrative and practical problems themselves. Working in an environment without peers can lead to isolation; it has been shown that face-to-face contact with colleagues had a positive impact on job satisfaction [25]. We found that the association between time spent on administrative work tasks and reported role conflicts was stronger among allied professionals than among PCPs.

A large proportion of work-time involved documentation of medical records, which is controlled by Swedish law [26]. Staff members only have a small amount of influence regarding this task. Part of the problem is that IT systems for health care documentation present many challenges [27–29], and most systems do not save time [30, 31]. However, IT systems can reduce the work burden for care administrators [31]. Care administrators spent a high proportion of time on indirect patient work tasks, including documentation in medical records. Thus, they were engaged in the work tasks expected in their profession. In Sweden, care administrators primarily assist PCPs; in contrast, RNs, NAs, and allied professionals must deal with their own documentation (e.g., medical records). Nevertheless, the time spent on administrative tasks was similar among all professions; this finding indicated that the administrative burden for PCPs was relatively high compared to other professionals.

Overall, staff members reported a low amount of role conflicts, which might indicate a feeling of performing meaningful work. Senior staff reported less role conflicts and stress compared to junior staff, which could be explained by their long experience and confidence in their professional roles, consistent with the results reported by Schmitz [32].

### Strengths and limitations

This study had several strengths. The high response rates strengthened the credibility of this study, and the variation in the primary care centre sizes made the sample representative of Swedish primary care. Although only 11 out of the 23 invited primary care centres agreed to participate in the study, the urban-rural distribution in the final sample was similar to that of all 23 centres. Therefore, we concluded that the final sample size was not likely to have affected the generalizability of our results to this geographical area. The design of the time-study (data were recorded every hour) ensured that the risk of recall bias was minimized [11]. The COPSOQ instrument has been validated, and its reliability was later confirmed in a Swedish context [33]. The study also had some limitations. The self-reporting method might have introduced some methodological challenges. The interpretation of the work task definitions may have varied among participants; e.g., it may have been difficult to distinguish between direct and indirect work tasks. To avoid confusion, participants were informed which work tasks comprised direct, indirect, and other work tasks. In addition, each participant received a pamphlet with instructions on how to complete the time-study form. We could not rule out the possibility that participants might not have always responded completely truthfully. To our knowledge, the time study, where individuals reported precise times spent on work tasks, was the most comprehensive study of its kind performed in Swedish primary care.

## Conclusions

This study indicated that Swedish primary care staff spent a limited proportion of their work time directly with patients. PCPs seemed to perceive their work environment in negative terms to a greater extent than other staff members. Allocation of time spent on work tasks influenced staff perceptions of the psychosocial work environment. Future research, possibly with a qualitative design, might shed further light on the results from this study and provide suggestions on how to improve the psychosocial work environment in primary care.

### Potential implications

The results of this study were not surprising, given the complex, bureaucratic organization in Swedish primary care. For more efficient use of work-time among medical staff in primary care, we recommend an increase in the number of administrative and service personnel.

## Additional files

Additional file 1: Time study data collection form. The file contains the form where the participants recorded the time (min) they spent on each work task, every hour, every day, over two separate weeks, Monday to Friday, during office hours. The form contained three main categories (called work tasks) and a number of subcategories for each main category. (DOCX 26 kb)

Additional file 2: Comparisons between professionals in COPSOQ scores. The means and SD of COPSOQ scales were compared between professions with the analysis of variance (ANOVA) and post-hoc Tukey test. (DOCX 18 kb)

## Abbreviations

COPSOQ: Copenhagen Psychosocial Questionnaire
NA: Nurse Assistant
PCP: Primary Care Physician
RN: Registered Nurse
