# Supplementary material for: Time utilization and perceived psychosocial work environment among staff in Swedish primary care settings
Source: BMC Health Serv Res. 2018 Mar 7;18:166. doi: 10.1186/s12913-018-2948-6 (PMC5842529; doi:10.1186/s12913-018-2948-6)
Supplement: Supplementary file 1 — Time study data collection form. The file contains the form where the participants recorded the time (min) they spent on each work task, every hour, every day, over two separate weeks, Monday to Friday, during office hours. The form contained three main categories (called work tasks) and a number of subcategories for each main category. (DOCX 26 kb) [file 12913_2018_2948_MOESM1_ESM.docx]

| **Additional file 1.** Time study data collection form | | | | | |  | |  | |  | |  | |  | |  |
| --- | --- | --- | --- | --- | --- | --- | --- | --- | --- | --- | --- | --- | --- | --- | --- | --- |
| Weekday | **Hour 1** | **Hour 2** | **Hour 3** | **Hour 4** | **Hour 5** | | **Hour 6** | | **Hour 7** | | **Hour 8** | | **Hour 9** | | **Hour >10** | |
| **Direct patient work tasks** |  |  |  |  |  | |  | |  | |  | |  | |  | |
| Face-to-face contact with patient | min | min | min | min | min | | min | | min | | min | | min | | min | |
| Telephone contact with patient | min | min | min | min | min | | min | | min | | min | | min | | min | |
| Telephone contact with patients’ next of kin | min | min | min | min | min | | min | | min | | min | | min | | min | |
| Remaining tasks: | min | min | min | min | min | | min | | min | | min | | min | | min | |
| **Indirect patient work tasks** |  |  |  |  |  | |  | |  | |  | |  | |  | |
| Reading health care records | min | min | min | min | min | | min | | min | | min | | min | | min | |
| Dictation | min | min | min | min | min | | min | | min | | min | | min | | min | |
| Documentation health care records, order tests | min | min | min | min | min | | min | | min | | min | | min | | min | |
| Referral management | min | min | min | min | min | | min | | min | | min | | min | | min | |
| Signing | min | min | min | min | min | | min | | min | | min | | min | | min | |
| Handle mailings | min | min | min | min | min | | min | | min | | min | | min | | min | |
| Prescribing medical drugs | min | min | min | min | min | | min | | min | | min | | min | | min | |
| Prescription of medical aids | min | min | min | min | min | | min | | min | | min | | min | | min | |
| Drug management | min | min | min | min | min | | min | | min | | min | | min | | min | |
| Administering appointments | min | min | min | min | min | | min | | min | | min | | min | | min | |
| Patient-related transport | min | min | min | min | min | | min | | min | | min | | min | | min | |
| Contact with other caregivers about patient cases | min | min | min | min | min | | min | | min | | min | | min | | min | |
| Contact with authorities | min | min | min | min | min | | min | | min | | min | | min | | min | |
| Reporting data into quality registries | min | min | min | min | min | | min | | min | | min | | min | | min | |
| Remaining tasks: | min | min | min | min | min | | min | | min | | min | | min | | min | |
| **Other work tasks** |  |  |  |  |  | |  | |  | |  | |  | |  | |
| Meetings at work place | min | min | min | min | min | | min | | min | | min | | min | | min | |
| Meetings outside work place | min | min | min | min | min | | min | | min | | min | | min | | min | |
| Continuing education | min | min | min | min | min | | min | | min | | min | | min | | min | |
| Receiving and giving mentoring | min | min | min | min | min | | min | | min | | min | | min | | min | |
| Non-patient-related telephone contacts | min | min | min | min | min | | min | | min | | min | | min | | min | |
| Non-patient-related transport | min | min | min | min | min | | min | | min | | min | | min | | min | |
| Managing e-mails | min | min | min | min | min | | min | | min | | min | | min | | min | |
| Scheduling | min | min | min | min | min | | min | | min | | min | | min | | min | |
| Ordering medical supplies, including laundry | min | min | min | min | min | | min | | min | | min | | min | | min | |
| Managing equipment and facilities, non-computer related | min | min | min | min | min | | min | | min | | min | | min | | min | |
| Managing computer problems | min | min | min | min | min | | min | | min | | min | | min | | min | |
| Waiting, non-computer related | min | min | min | min | min | | min | | min | | min | | min | | min | |
| Other writing tasks/administration | min | min | min | min | min | | min | | min | | min | | min | | min | |
| Pauses | min | min | min | min | min | | min | | min | | min | | min | | min | |
| Lunch | min | min | min | min | min | | min | | min | | min | | min | | min | |
| Remaining tasks: | min | min | min | min | min | | min | | min | | min | | min | | min | |
| TOTALLY | **60 min** | **60 min** | **60 min** | **60 min** | **60 min** | | **60 min** | | **60 min** | | **60 min** | | **60 min** | | **min** | |
