# Supplementary material for: Time utilization and perceived psychosocial work environment among staff in Swedish primary care settings
Source: BMC Health Serv Res. 2018 Mar 7;18:166. doi: 10.1186/s12913-018-2948-6 (PMC5842529; doi:10.1186/s12913-018-2948-6)
Supplement: Supplementary file 2 — Comparisons between professionals in COPSOQ scores. The means and SD of COPSOQ scales were compared between professions with the analysis of variance (ANOVA) and post-hoc Tukey test. (DOCX 18 kb) [file 12913_2018_2948_MOESM2_ESM.docx]

**Additional file 2.** Comparisons between professionals in COPSOQ scores

|  |  |  |  | P-values for pairwise comparisons^a^ | | | | | | | | | | |
| --- | --- | --- | --- | --- | --- | --- | --- | --- | --- | --- | --- | --- | --- | --- |
| Professional |  | Professional |  | Quantitative demands |  | Stress |  | Role conflicts |  | Quality in work |  | Conflicts between work and personal life |  | Positive impact of work to personal life |
| Registered nurse | vs. | Care administrator |  | 0.789 |  | 1.000 |  | 1.000 |  | 0.959 |  | 0.093 |  | 0.523 |
| Registered nurse | vs. | Nurse assistant |  | 0.005 |  | 0.707 |  | 0.954 |  | 0.782 |  | 0.054 |  | 1.000 |
| Registered nurse | vs. | Allied professionals |  | 0.855 |  | 0.998 |  | 1.000 |  | 0.986 |  | 0.981 |  | 0.797 |
| Primary care physician | vs. | Registered nurse |  | <0.001 |  | 0.011 |  | <0.001 |  | 0.988 |  | <0.001 |  | 0.997 |
| Primary care physician | vs. | Care administrator |  | <0.001 |  | 0.057 |  | 0.002 |  | 1.000 |  | <0.001 |  | 0.465 |
| Primary care physician | vs. | Nurse assistant |  | <0.001 |  | 0.004 |  | 0.002 |  | 0.960 |  | <0.001 |  | 0.992 |
| Primary care physician | vs. | Allied professionals |  | 0.093 |  | 0.184 |  | 0.008 |  | 1.000 |  | 0.003 |  | 0.945 |
| Care administrator | vs. | Nurse assistant |  | 0.135 |  | 0.718 |  | 0.976 |  | 0.984 |  | 0.971 |  | 0.873 |
| Care administrator | vs. | Allied professionals |  | 0.397 |  | 1.000 |  | 1.000 |  | 1.000 |  | 0.117 |  | 0.185 |
| Allied professionals | vs. | Nurse assistant |  | 0.003 |  | 0.705 |  | 0.987 |  | 0.984 |  | 0.061 |  | 0.831 |

^a^P-values were based on ANOVA and post-hoc-test Tukey; columns indicate results for individual dimensions in the COPSOC questionnaire
